# The impact of physical activity variability with the risk of hypertension: Insights from a national longitudinal study

**DOI:** 10.1097/MD.0000000000044289

**Published:** 2025-10-03

**Authors:** Shan Chen, Chunmei Liu, Juan Huang, Huaying Deng, Jie Cao, Meng Jia, Qinke Li

**Affiliations:** a Inpatient Department, Qian Feng Hospital, Chengdu, Sichuan, China; b Department of Cardiovascular Medicine, National Nuclear Corporation 416 Hospital, The Second Affiliated Hospital of Chengdu Medical College, Chengdu, Sichuan, China; c Department of Basic, Luzhou College of Medical Devices, Luzhou, Sichuan, China.

**Keywords:** China, cohort study, hypertension, physical activity variability

## Abstract

This study evaluates the relationship between physical activity (PA) variability (PAVar) and the risk of hypertension in middle-aged and older adults. This longitudinal cohort study utilized data from the China Health and Retirement Longitudinal Study, spanning from 2011 to 2020. A total of 4870 participants with complete PA data were categorized into quartiles based on the coefficient of variation for PAVar. Hypertension incidence was assessed through self-reported physician diagnosis, blood pressure measurements, and antihypertensive medication use. Cox proportional hazards models, adjusted for demographic, socioeconomic, and lifestyle factors, were employed to estimate hazard ratios. Mediation analysis examined the potential role of sleep duration in the PAVar-hypertension relationship, and sensitivity analyses excluded participants with missing baseline data to ensure robustness. Higher PAVar was associated with increased hypertension risk. In fully adjusted models, participants in the highest coefficient of variation quartile had a 75% higher risk of hypertension (HR: 1.75, 95% CI: 1.57–1.95) compared to the lowest quartile. Sleep duration mediated 26.3% of the total effect of PAVar on hypertension risk. Sensitivity analyses confirmed the stability of these findings. This study identifies a significant association between high PAVar and elevated hypertension risk, emphasizing the importance of consistent PA and adequate sleep for hypertension prevention. These findings provide evidence to support tailored public health strategies for hypertension management in aging populations.

## 1. Introduction

Hypertension, or high blood pressure, is a leading global public health issue, often termed a “silent killer” due to its asymptomatic nature in many cases.^[1]^ It remains a primary risk factor for cardiovascular diseases, kidney disease, and premature mortality worldwide.^[2,3]^ According to the World Health Organization, approximately 1.3 billion adults globally were living with hypertension in 2019, with low- and middle-income countries bearing the majority of the burden.^[4,5]^ Alarmingly, over half of these individuals remain undiagnosed, untreated, or inadequately controlled.^[6,7]^ The disease contributes significantly to the global disease burden, accounting for more than 10 million deaths annually, primarily through heart attacks, strokes, and other cardiovascular complications.^[8,9]^

The prevalence of hypertension has been increasing in China, mirroring global trends.^[10]^ National surveys reveal that the prevalence of hypertension among Chinese adults rose from 23.2% in 2015 to 27.5% in 2018, highlighting a growing public health challenge.^[11,12]^ Even among children and adolescents, a troubling increase has been observed, with prevalence reaching 13.0% in 2019, pointing to early onset and potentially lifelong implications of the condition.^[13,14]^ This upward trend necessitates the identification of modifiable risk factors to inform prevention and management strategies.

Physical activity (PA) is widely regarded as one of the most effective and modifiable factors in managing blood pressure and reducing the risk of hypertension.^[15,16]^ Regular moderate-to-vigorous physical activity (PA) improves vascular health by enhancing endothelial function, reducing arterial stiffness, and promoting better weight and stress management.^[17,18]^ However, despite well-established benefits, real-world physical activity patterns often show significant fluctuations due to lifestyle, occupational demands, and seasonal factors.^[19]^ These variations, collectively termed physical activity variability (PAVar), have recently garnered attention for their potential impact on metabolic and cardiovascular health.^[11,20]^

Emerging evidence suggests that irregular PA patterns may disrupt physiological homeostasis, leading to adverse health outcomes. For instance, variability in exercise habits can interfere with circadian rhythms, insulin sensitivity, and autonomic regulation, all of which are critical in maintaining normal blood pressure.^[21,22]^ However, most studies examining PA and hypertension focus on total activity levels rather than consistency over time. This gap in knowledge highlights the need for research into how PAVar influences hypertension risk.

This study seeks to address this knowledge gap by investigating the relationship between PAVar and hypertension using data from a national prospective cohort. By analyzing longitudinal data, we aim to elucidate whether greater variability in PA increases the risk of hypertension, independent of total activity levels.^[23,24]^ Furthermore, we will explore potential mediators, such as sleep quality and stress, that may link PAVar with blood pressure regulation. Understanding these associations can inform tailored public health interventions to promote consistent PA patterns for hypertension prevention and management.

## 2. Methodology

### 2.1. Data source and ethical considerations

This research utilized data from the China Health and Retirement Longitudinal Study, a large-scale, nationally representative survey that provides extensive insights into the health, social, and economic status of middle-aged and elderly populations in China. To achieve comprehensive geographic and demographic representation across 28 provinces, China Health and Retirement Longitudinal Study implemented a multi-stage, stratified sampling methodology. Initial data collection commenced in 2011, with subsequent follow-up waves conducted in 2013, 2015, 2018, and 2020. The study protocol was reviewed and approved by the Institutional Review Board of Peking University (IRB00001052-11015), and all participants provided written informed consent prior to participation.^[25]^

### 2.2. Study participants

**Figure 1.**
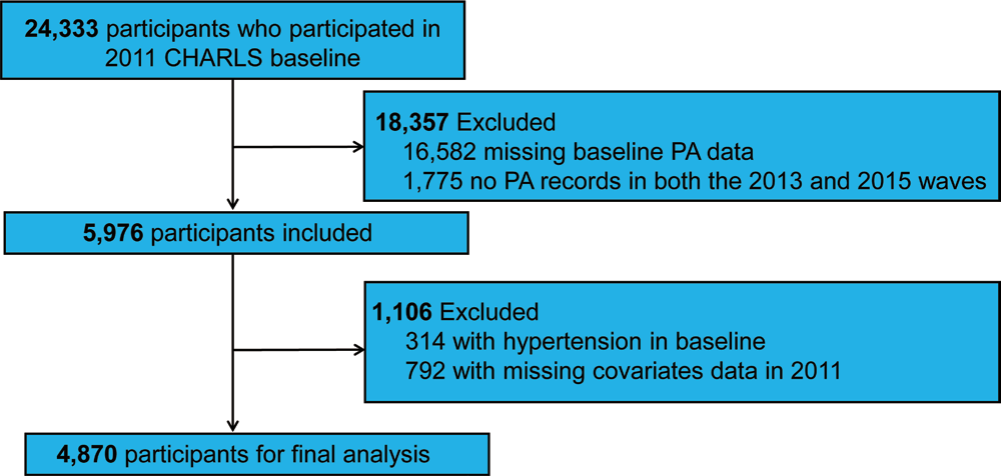
Flowchart of participant selection.

The original dataset consisted of 24,333 individuals surveyed in 2011. To be eligible for inclusion, participants needed to have complete PA data at baseline, participate in at least 1 follow-up wave, and have no history of hypertension diagnosis. Sequential exclusions were applied as follows: individuals missing baseline PA data (n = 16,582), those with no PA records in both the 2013 and 2015 waves (n = 1775), participants reporting a prior diagnosis of hypertension or with baseline systolic blood pressure ≥ 140 mm Hg and/or diastolic blood pressure ≥90 mm Hg (n = 314), and cases with missing demographic or covariate data required for multivariable adjustments (n = 792). After applying these criteria, the final analytical sample included 4870 participants, as depicted in the selection process flowchart (Fig. 1).

### 2.3. Measuring physical activity

PA assessment was conducted using a validated questionnaire derived from the International PA Questionnaire. Participants provided details on the frequency and average daily duration of activities lasting at least 10 minutes, which were classified into 3 intensity categories: vigorous (heavy lifting, digging, or aerobic exercises), moderate (brisk walking, tai chi, or regular cycling), and light (casual walking or household chores). To standardize activity durations, reported time intervals were converted to weekly totals using midpoint estimates: 10 to 30 minutes as 20 minutes, 30 to 120 minutes as 75 minutes, 120 to 240 minutes as 180 minutes, and over 240 minutes as 240 minutes. The total PA volume (PAV) was then quantified in metabolic equivalent of task (MET) minutes per week using the formula: PAV = (8.0 × vigorous activity) + (4.0 × moderate activity) + (3.3 × light activity).

### 2.4. Quantifying PAVar

PA variability was evaluated using the Coefficient of Variation (CV), a measure that represents the relative fluctuations in activity levels over time. The CV for each participant was computed using the formula:


$$\begin{array}{lll} CV= & (Standard\;deviation\;of\;PAV\;across\;waves\; & /Mean\;PAV\;across\;waves)\times 100\% \end{array}$$

This metric was based on PA data collected from the 2011, 2013, and 2015 survey waves. To investigate the relationship between variability in PA and hypertension risk, participants were categorized into quartiles according to their CV values.

### 2.5. Hypertension diagnosis and follow-up

The primary outcome was the onset of hypertension, determined through the following criteria: Self-reported diagnosis by a physician during follow-up. Systolic blood pressure ≥ 140 mm Hg and/or diastolic blood pressure ≥90 mm Hg in follow-up surveys. Use of antihypertensive medications was reported during follow-up. Participants were monitored until the first occurrence of hypertension diagnosis, death, loss to follow-up, or the conclusion of the 2020 survey wave. Time-to-event data were recorded to support survival analysis.

### 2.6. Covariates and confounder adjustment

The analysis accounted for a range of potential confounders to ensure robust results.^[26]^ Specifically, we considered the following categories and variables:

1. Demographic factors: age (continuous, in years), gender (male/female), marital status (married vs unmarried), residence (urban vs rural), and educational level (illiterate, primary school and below, middle/high school, technical school and above)
2. Socioeconomic factors: employment status (no job, agricultural job, nonagricultural job), household income (non-income, low, middle, high), medical insurance (urban employee medical insurance, urban and rural resident medical insurance, urban resident medical insurance, new cooperative medical insurance, or no insurance)
3. Lifestyle habits: smoking status (never smoking, former smoking, still smoking), alcohol use (current drinking vs not currently drinking)
4. Health-related indicators: sleep duration (continuous, hours), body mass index (continuous; kg/m^2^), presence of dyslipidemia in baseline (yes or no), presence of diabetes in baseline (yes or no), and presence of kidney disease in baseline (yes or no).

### 2.7. Statistical analysis

Descriptive statistics were employed to outline the baseline characteristics of the study population across quartiles of the CV for PAVar. Continuous variables were summarized using means with standard deviations or medians with interquartile ranges (IQR), depending on the data distribution, while categorical variables were reported as frequencies and percentages. Comparisons among CV quartiles were conducted using one-way analysis of variance (ANOVA) or the Kruskal–Wallis test for continuous variables and chi-square tests for categorical variables.

The primary outcome, the incidence of hypertension, was evaluated using Cox proportional hazards regression to calculate hazard ratios with 95% confidence intervals for each CV quartile, using the lowest quartile as the reference. Specifically, we conducted both global and covariate‐specific tests: the global test yielded χ² = 2.13 (*df *= 3), *P *= .548, and tests for individual quartiles of PAVar_CV gave *P* = .672 (Q2), *P* = .481 (Q3), and *P* = .391 (Q4). No significant violations were detected, supporting the validity of our Cox models. Four models were constructed to address confounding factors progressively: Model I was unadjusted (crude model); Model II included adjustments for all demographic factors; and Model III incorporated additional adjustments for socioeconomic factors and lifestyle habits; Model IV is a fully adjusted model based on Model III, with additional adjustments for all health-related indicators and PAV.

To explore the dose-response relationship between CV and hypertension risk, restricted cubic spline regression was applied, offering a flexible method to identify nonlinear associations. Schoenfeld residuals were used to confirm the proportional hazards assumptions, with no significant violations detected. Additionally, mediation analysis was conducted to evaluate whether sleep duration played a mediating role in the relationship between PA variability (PAVar_CV) and hypertension risk. The total effect of PAVar_CV on hypertension risk was decomposed into direct and indirect effects mediated by sleep duration. Structural equation modeling and bootstrap methods were employed to estimate and test the significance of these effects.^[27]^

A sensitivity analysis was also performed to validate the robustness of the findings by excluding participants with missing baseline data on key variables, including age group, gender, education level, marital status, medical insurance, smoking status, alcohol consumption, income category, and employment status. This analysis assessed how these exclusions influenced the hazard ratio estimates for hypertension. All statistical procedures were conducted using Stata 16.0 (StataCorp, College Station, TX, USA) and R version 4.3.2. A 2-sided *P*-value of <.05 was considered to indicate statistical significance in all analyses.

## 3. Results

### 3.1. Characteristics for baseline participants

The baseline characteristics of the 4870 participants, divided into quartiles based on the CV for PAVar, are detailed in Table 1. Participants’ ages ranged from 45 to 118 years, with a mean of 61.5 ± 9.2 years. Of the 4870 participants included in our final analysis, a total of 354 individuals (7.3%) were lost to follow-up, and 151 (3.1%) were dead before reaching the final survey wave in 2020. The mean follow-up time for the remaining participants was 7.2 ± 2.1 years. Participants in the highest variability quartile (Q4) were older (62.8 ± 9.5 vs 60.2 ± 8.5 years in Q1; *P* <.001) and included more women (66.3% vs 60.3%; *P* = .037). Educational level, marital status, household income, residence, medical insurance coverage, smoking status, and alcohol use did not differ significantly across quartiles (all *P* >.05). Employment patterns varied markedly (*P* <.001): agricultural work declined from 61.6% in Q1 to 30.6% in Q4, nonagricultural employment rose from 9.6% to 34.0%, and “no job” increased from 28.8% to 35.3%. Dyslipidemia prevalence also increased (12.3% to 20.8%; *P *<.001), whereas diabetes and kidney disease remained stable (*P* >.05). Mean body mass index and sleep duration showed nonsignificant trends (23.4 ± 4.6 to 23.9 ± 4.2 kg/m²; 7.5 ± 1.4 to 7.0 ± 1.5 hours; *P* >.05). In addition, total PAV showed only modest differences across variability quartiles, with an overall mean of 6345.9 ± 6369.9 MET-minutes/week; by quartile: Q1 5800.2 ± 5912.4, Q2 6712.5 ± 6502.1, Q3 6102.8 ± 6354.9, and Q4 7115.7 ± 7021.3 MET-minutes/week. Finally, median PAVar_CV rose sharply from 15.5 (IQR 7.7–27.0) in Q1 to 127.5 (IQR 113.0–142.0) in Q4 (*P* <.001).

**Table 1:** Baseline characteristics of participants grouped by PA variability.

Variables	Total	Q1	Q2	Q3	Q4
	(N = 3970)	(N = 987)	(N = 1000)	(N = 995)	(N = 988)
Age (years)	61.5 ± 9.2	60.2 ± 8.5	61.1 ± 8.8	62.1 ± 9.2	62.8 ± 9.5
Gender					
Female, N (%)	2495 (62.83)	595 (60.28)	615 (61.50)	630 (63.32)	655 (66.30)
Male, N (%)	1475 (37.17)	392 (39.72)	385 (38.50)	365 (36.68)	333 (33.70)
Educational level					
Illiterate, N (%)	281 (7.09)	65 (6.59)	70 (7.00)	74 (7.44)	72 (7.29)
Primary school and below, N (%)	358 (9.03)	81 (8.21)	90 (9.00)	86 (8.64)	101 (10.22)
Middle school and high school, N (%)	191 (4.82)	49 (4.96)	45 (4.50)	49 (4.92)	48 (4.86)
Technical school and above, N (%)	3140 (79.06)	792 (80.24)	795 (79.50)	786 (79.00)	767 (77.56)
Marital status					
Married, N (%)	3479 (87.66)	889 (90.06)	879 (87.90)	870 (87.44)	841 (85.12)
Unmarried, N (%)	491 (12.34)	98 (9.94)	121 (12.10)	125 (12.56)	147 (14.88)
Residence					
Rural, N (%)	2792 (70.33)	711 (71.92)	706 (70.60)	695 (69.85)	680 (68.83)
Urban, N (%)	1178 (29.67)	276 (28.00)	294 (29.40)	300 (30.15)	308 (31.17)
Employment status					
No job, N (%)	1272 (32.02)	284 (28.76)	308 (30.80)	331 (33.29)	349 (35.32)
Agricultural job, N (%)	1849 (46.61)	608 (61.62)	538 (53.80)	401 (40.30)	302 (30.57)
Nonagricultural job, N (%)	849 (21.37)	95 (9.62)	154 (15.40)	263 (26.43)	337 (34.03)
Household income					
Non-income, N (%)	2847 (71.77)	722 (73.17)	726 (72.60)	702 (70.55)	697 (70.55)
Low income, N (%)	94 (2.36)	24 (2.43)	21 (2.10)	25 (2.51)	24 (2.43)
Middle income, N (%)	559 (14.06)	134 (13.57)	140 (14.00)	143 (14.37)	142 (14.37)
High income, N (%)	470 (11.82)	107 (10.84)	113 (11.30)	125 (12.56)	125 (12.66)
Medical insurance					
UEMI, N (%)*	292 (7.36)	72 (7.30)	74 (7.40)	73 (7.34)	73 (7.39)
URRMI, N (%)*	70 (1.76)	17 (1.72)	18 (1.80)	17 (1.71)	18 (1.82)
URMI, N (%)*	148 (3.73)	37 (3.75)	37 (3.70)	37 (3.72)	37 (3.74)
NCMI, N (%)*	3222 (81.16)	802 (81.27)	811 (81.10)	808 (81.21)	801 (81.05)
No insurance, N (%)	238 (5.99)	59 (5.98)	60 (6.00)	60 (6.03)	59 (5.97)
Smoking status					
Never smoking, N (%)	1952 (49.17)	491 (49.71)	501 (50.10)	482 (48.44)	478 (48.38)
Former smoking, N (%)	767 (19.33)	195 (19.75)	201 (20.10)	204 (20.50)	167 (16.90)
Still smoking, N (%)	1251 (31.51)	301 (30.50)	298 (29.80)	309 (31.06)	343 (34.72)
Alcohol use					
Current drinking, N (%)	1492 (37.56)	369 (37.40)	382 (38.20)	372 (37.39)	369 (37.35)
Not current drinking, N (%)	2478 (62.44)	618 (62.60)	618 (61.80)	623 (62.61)	619 (62.65)
Dyslipidemia					
Yes, N (%)	607 (15.29%)	121 (12.26%)	127 (12.70%)	153 (15.38%)	206 (20.85%)
No, N (%)	3363 (84.71%)	866 (87.74%)	873 (87.30%)	842 (84.62%)	782 (79.15%)
Diabetes					
Yes, N (%)	412 (10.38%)	104 (10.54%)	102 (10.20%)	99 (9.95%)	107 (10.83%)
No, N (%)	3558 (89.62%)	883 (89.46%)	898 (89.80%)	896 (90.05%)	881 (89.17%)
Kidney disease					
Yes, N (%)	198 (5.00)	49 (4.96)	50 (5.00)	50 (5.03)	49 (4.96)
No, N (%)	3772 (95.00)	938 (95.04)	950 (95.00)	945 (94.97)	939 (95.04)
BMI (kg/m²)	23.6 ± 4.4	23.4 ± 4.6	23.5 ± 4.3	23.7 ± 4.4	23.9 ± 4.2
Sleep duration (h)	7.2 ± 1.5	7.5 ± 1.4	7.3 ± 1.5	7.1 ± 1.6	7.0 ± 1.5
PAV (MET-min/wk)	6345.9 ± 6369.9	5800.2 ± 5912.4	6712.5 ± 6502.1	6102.8 ± 6354.9	7115.7 ± 7021.3
PAVar_CV (%)	68.5 (31.5–117.0)	15.5 (7.7–27.0)	45.0 (39.0–54.5)	78.0 (70.0–87.5)	127.5 (113.0–142.0)

BMI = body mass index, MET = metabolic equivalent of task, NCMI = New Cooperative Medical Insurance, PA = physical activity, PAV = physical activity volume, PAVar_CV = coefficient of variation for physical activity variability, Q = quartile, SD = standard deviation, UEMI = Urban Employee Medical Insurance, URRMI = Urban and Rural Resident Medical Insurance, URMI = Urban Resident Medical Insurance.

* Data are presented as mean ± SD, median (interquartile range), or N (%).

### 3.2. Incidence of hypertension according to PPV

**Figure 2.**
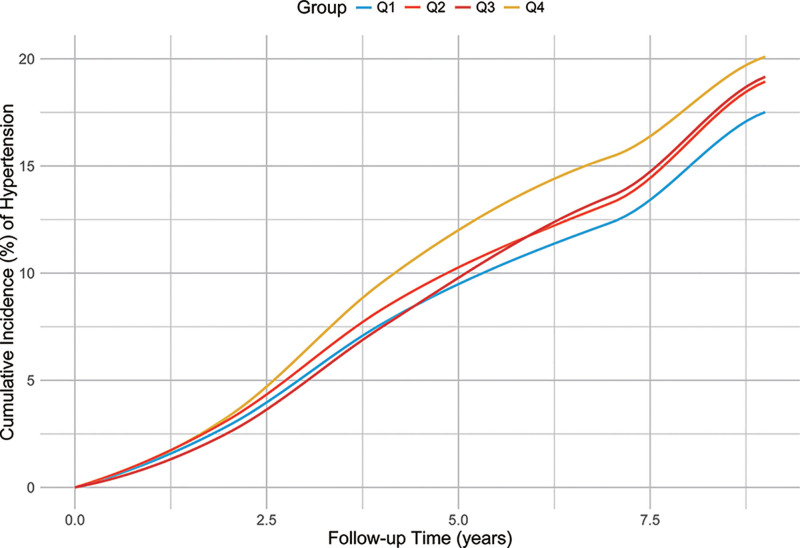
Cumulative incidence of hypertension according to PAVar_CV. PAVar_CV = coefficient of variation for physical activity variability

**Figure 3.**
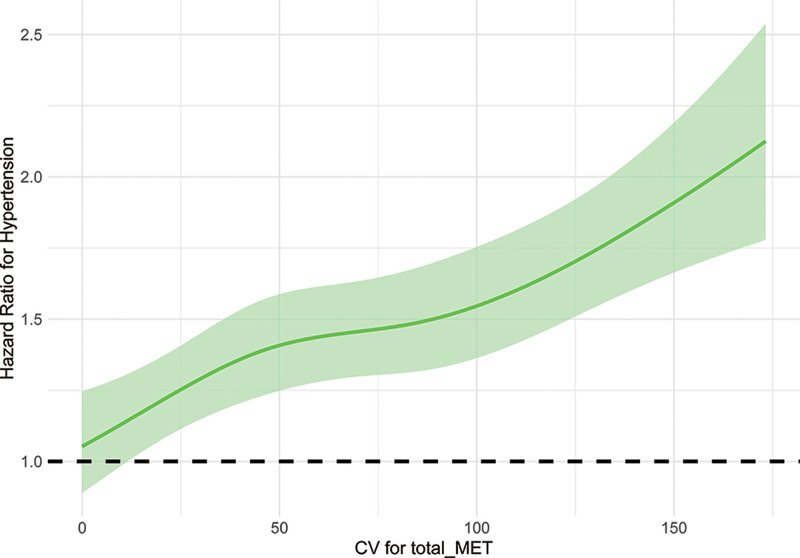
Dose-response relationship between PAVar_CV and hypertension among Chinese middle-aged and older adults. PAVar_CV = coefficient of variation for physical activity variability

Table 2 provides the hazard ratios and 95% confidence intervals for hypertension risk across quartiles of PAVar_CV. The findings indicate a clear upward trend in hypertension risk with increasing variability. In the fully adjusted model (Model IV), participants in the highest quartile (Q4) exhibited the greatest risk (HR: 1.72, 95% CI: 1.54–1.92) relative to those in the lowest quartile (Q1, reference). Incidence rates also followed a similar pattern, rising from 14.2 per 1000 person-years in Q1 to 26.3 per 1000 person-years in Q4. The trend across quartiles was highly significant (*P* for trend <.001), reinforcing the association between higher variability in PA and an elevated risk of hypertension. These results underscore the importance of maintaining consistent PA levels to mitigate hypertension risk. Figure 2 shows the cumulative incidence of hypertension according to PAVar_CV. Figure 3 shows the dose-response relationship between PAVar_CV and hypertension among Chinese middle-aged and older adults.

**Table 2:** Association between PAVar_CV and risk of hypertension.

Variables	Participants	Events	Incidence rate	Model I	Model II	Model III	Model IV
–	(N)	(n)	(per1000 PYs)	HR (95% CI)	HR (95% CI)	HR (95% CI)	HR (95% CI)
PAVar_CV Q1	1217	105	14.2	Ref	Ref	Ref	Ref
Q2	1218	140	17.5	1.30 (1.15–1.47)	1.27 (1.13–1.43)	1.22 (1.10–1.36)	1.18 (1.06–1.32)
Q3	1218	175	22	1.60 (1.42–1.80)	1.57 (1.39–1.76)	1.52 (1.35–1.70)	1.46 (1.28–1.66)
Q4	1217	200	26.3	1.90 (1.70–2.12)	1.82 (1.64–2.04)	1.77 (1.59–1.97)	1.72 (1.54–1.92)
*P* for trend	–	–	–	<.001	<.001	<.001	<.001

CI = confidence interval, HR = hazard ratio, PAV = physical activity volume, PAVar_CV = coefficient of variation for physical activity variability, Q1–Q4 = quartiles 1 through 4.

Model I was unadjusted (crude model); Model II included adjustments for all demographic factors; and Model III incorporated additional adjustments for socioeconomic factors and lifestyle habits; Model IV is a fully adjusted model based on Model III, with additional adjustments for all health-related indicators and PAV.

### 3.3. Mediation analysis

**Figure 4.**
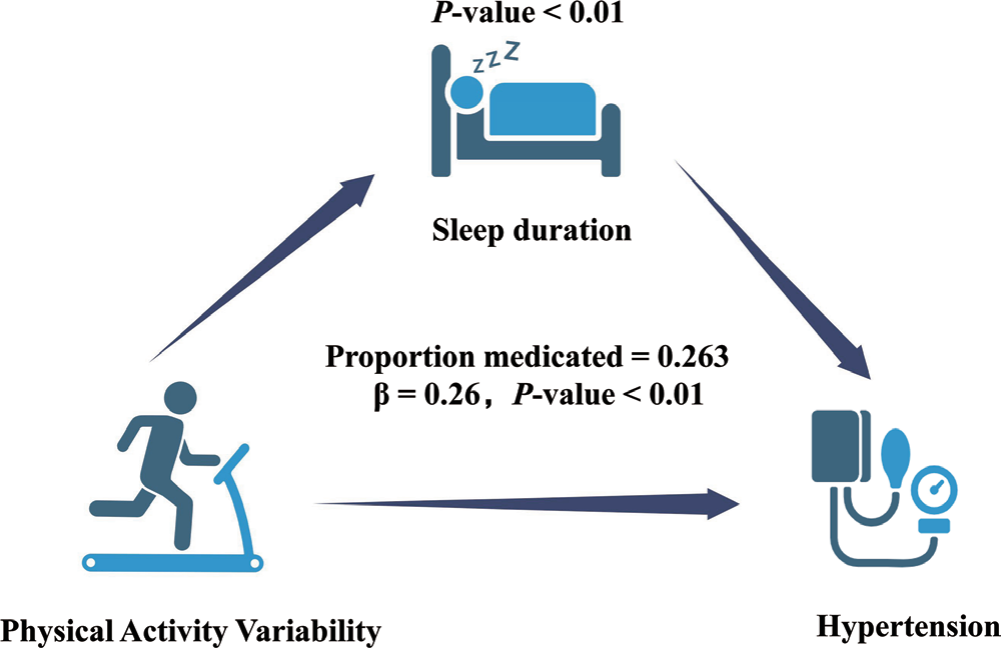
Path diagram of mediation analysis of relationship between PAVar_CV, sleep duration, and hypertension. PAVar_CV = coefficient of variation for physical activity variability.

The mediation analysis demonstrated that sleep duration played a significant mediating role in the relationship between PAVar_CV and hypertension risk (Fig. 4). Sleep duration was found to account for 26.3% of the overall effect of PAVar_CV on hypertension risk (indirect effect: β = 0.26, *P *= .02), with the mediation effect reaching statistical significance. Conversely, sleep duration did not show significant mediation effects in the associations between PAVar_CV and other variables, such as income level and hypertension risk.

### 3.4. Sensitivity analysis

To verify the reliability of the findings, a sensitivity analysis was carried out by excluding participants with incomplete baseline data on all confounders. The results showed that these exclusions had little to no impact on the estimated risk for hypertension, as all *P*-value differences remained above 0.05, confirming the stability of the conclusions (Appendix, Supplemental Digital Content, https://links.lww.com/MD/P955).

## 4. Discussion

This study investigated the association between PAVar and the risk of hypertension among middle-aged and older adults in China. The findings showed that higher PAVar was significantly associated with an increased risk of hypertension. Participants in the highest quartile of PAVar had a 72% greater risk of developing hypertension compared to those in the lowest quartile (HR: 1.72; 95% CI: 1.54–1.92), even after adjusting for demographic, socioeconomic, and lifestyle factors. Mediation analysis revealed that sleep duration partially mediated this relationship, accounting for 26.3% of the total effect, highlighting the importance of consistent PA patterns and adequate sleep in preventing hypertension.

When comparing our findings to previous research, this study expands on existing knowledge by shifting the focus from total PAV to its variability.^[9,13,28]^ While prior studies have consistently demonstrated the protective effects of regular PA on hypertension, the impact of variability in PA remains less understood. For example, a U.S.-based cohort study highlighted that consistent moderate-to-vigorous PA reduced hypertension risk by 21% over 5 years, but did not examine the effects of activity patterns.^[29]^ Our findings align with recent research suggesting that fluctuations in lifestyle behaviors, such as diet and PA, may exacerbate cardiovascular risks, emphasizing the need for stability in health behaviors across populations. However, cultural differences, such as dietary habits and work-related PA, suggest that further validation in diverse settings is warranted.^[30,31]^

The clinical significance of this study lies in its potential to guide public health interventions and personalized treatment strategies.^[25]^ By demonstrating the detrimental effects of PA variability, this study underscores the importance of not only promoting PA but also encouraging consistency in exercise patterns. Targeted interventions, such as behavioral counseling or digital health tools that monitor and stabilize PA patterns, could be developed to mitigate hypertension risk in vulnerable populations.^[32,33]^ Moreover, the identification of sleep duration as a mediator highlights an actionable pathway that clinicians can address to optimize cardiovascular health. These findings contribute to a growing body of evidence that supports a holistic approach to hypertension prevention, integrating both PA management and sleep hygiene.^[34,35]^

From a biomedical perspective, irregular PA can disrupt circadian rhythms and impair autonomic nervous system regulation, contributing to adverse cardiovascular outcomes.^[11,23,36]^ Studies have shown that variability in PA is linked to increased blood pressure variability, which is a well-established predictor of cardiovascular events and mortality.^[26,37,38]^ For instance, a study reported that a 10% increase in blood pressure variability raised the risk of cardiovascular death by 12% (95% CI: 6%–18%).^[12,23,39]^ Furthermore, irregular activity may lead to endothelial dysfunction and increased arterial stiffness, both of which are central mechanisms in the development of hypertension.^[40,41]^ These physiological disruptions provide a plausible explanation for the observed link between high PAVar and hypertension risk.^[32,42]^

The strengths of this study lie in its longitudinal design, enabling temporal relationships to be established, and its use of a large, nationally representative sample, enhancing the generalizability of the findings to middle-aged and older adults in China. The adjustment for a wide range of confounders and inclusion of sensitivity and mediation analyses further support the robustness of the results. However, certain limitations should be acknowledged.^[43,44]^ First, PA was self-reported, which may introduce recall bias. Second, the observational design precludes establishing causation, and residual confounding cannot be ruled out. Third, while sleep duration was examined as a mediator, other factors, such as stress levels and dietary intake, were not included in this analysis. Future studies should incorporate objective measures of PA and explore additional pathways linking PAVar to hypertension risk.^[45,46]^

## 5. Conclusion

This study demonstrates a strong association between PAVar and increased hypertension risk among middle-aged and older adults in China, with sleep duration partially mediating this relationship. The findings highlight the importance of consistent PA and adequate sleep in reducing hypertension risk. These results provide valuable insights for public health strategies and clinical interventions aimed at mitigating hypertension, emphasizing the need for tailored approaches to address variability in PA patterns. Future research should incorporate objective activity measures and explore additional mediators to validate these findings across diverse populations.

## Acknowledgments

The authors express their heartfelt appreciation to all participants of the study and gratefully acknowledge the support of the research team.

## Author contributions

**Conceptualization:** Shan Chen, Juan Huang.

**Methodology:** Juan Huang.

**Supervision:** Juan Huang.

**Funding acquisition:** Juan Huang.

**Data curation:** Shan Chen, Chunmei Liu, Huaying Deng, Meng Jia, Qinke Li.

**Formal analysis:** Shan Chen, Chunmei Liu.

**Investigation:** Jie Cao.

**Writing – original draft:** Shan Chen.

**Writing – review & editing:** Shan Chen, Chunmei Liu, Juan Huang, Huaying Deng, Jie Cao, Meng Jia, Qinke Li.

## Supplementary Material
