# Supplementary material for: The impact of physical activity variability with the risk of hypertension: Insights from a national longitudinal study
Source: Medicine (Baltimore). 2025 Oct 3;104(40):e44289. doi: 10.1097/MD.0000000000044289 (PMC12499712; doi:10.1097/MD.0000000000044289)
Supplement: Supplementary file 1 [file medi-104-e44289-s001.docx]

Sensitivity Analysis: association between PAVar_CV and risk of Hypertension

| Variables | Model I | Model II | Model III | Model IV | *P*_diff^*^ |
| --- | --- | --- | --- | --- | --- |
|  | HR (95%CI) | HR (95%CI) | HR (95%CI) | HR (95%CI) |  |
| PAVar_CV Q1 | Ref | Ref | Ref | Ref |  |
| Q2 | 1.29 (1.14–1.46) | 1.26 (1.12–1.42) | 1.21 (1.09–1.35) | 1.17 (1.05–1.31) | 0.18 |
| Q3 | 1.58 (1.40–1.78) | 1.55 (1.38–1.74) | 1.50 (1.34–1.68) | 1.44 (1.27–1.64) | 0.22 |
| Q4 | 1.88 (1.68–2.11) | 1.80 (1.62–2.02) | 1.75 (1.57–1.95) | 1.70 (1.52–1.90) | 0.16 |
| *P* for trend | <0.001 | <0.001 | <0.001 | <0.001 |  |

**P*_diff: *P*-value for difference between sensitivity and main analysis HR; >0.05, confirming robustness. Model I was unadjusted (crude model); Model II included adjustments for all demographic factors; Model III incorporated additional adjustments for socioeconomic factors and lifestyle habits; Model IV is a fully adjusted model based on Model III, with additional adjustments for all health-related indicators and PAV.
